# Room-temperature lasing from nanophotonic topological cavities

**DOI:** 10.1038/s41377-020-00350-3

**Published:** 2020-07-20

**Authors:** Daria Smirnova, Aditya Tripathi, Sergey Kruk, Min-Soo Hwang, Ha-Reem Kim, Hong-Gyu Park, Yuri Kivshar

**Affiliations:** 1 Nonlinear Physics Center, Research School of Physics, Australian National University, Canberra, ACT 2601 Australia; 2 Institute of Applied Physics, Russian Academy of Science, Nizhny Novgorod, 603950 Russia; 3 Department of Physics, Indian Institute of Technology, Delhi, 110016 India; 4 Department of Physics, Korea University, Seoul, 02841 Republic of Korea

**Keywords:** Lasers, LEDs and light sources, Nanophotonics and plasmonics

## Abstract

The study of topological phases of light underpins a promising paradigm for engineering disorder-immune compact photonic devices with unusual properties. Combined with an optical gain, topological photonic structures provide a novel platform for micro- and nanoscale lasers, which could benefit from nontrivial band topology and spatially localized gap states. Here, we propose and demonstrate experimentally active nanophotonic topological cavities incorporating III–V semiconductor quantum wells as a gain medium in the structure. We observe room-temperature lasing with a narrow spectrum, high coherence, and threshold behaviour. The emitted beam hosts a singularity encoded by a triade cavity mode that resides in the bandgap of two interfaced valley-Hall periodic photonic lattices with opposite parity breaking. Our findings make a step towards topologically controlled ultrasmall light sources with nontrivial radiation characteristics.

## Topological photonics: integrated nanolasers

Active topological cavities that can lase at room temperature could bring new opportunities for controlling light in integrated nanophotonic circuits. Smirnova et al. etched a special pattern of nanoscale holes into a 250 nm thick slab of the compound semiconductor InGaAsP to create a triangle-shaped cavity with optical behaviour governed by the band topology. The presence of quantum wells in the slab provides the cavity with optical gain allowing it to lase in the near-infrared when excited with nanosecond pump pulses at 980 nm. The laser emission is observed to be spectrally narrow with high coherence and hosts a donut-shaped singularity in Fourier space. The achievement opens the way to a new type of nanophotonic light source which exhibits unusual radiation characteristics and suits integration with metasurfaces.

## Introduction

Topological phases of light provide unique opportunities to create photonic systems immune to scattering losses and disorder^1^. In this scope, topologically robust wave transport typically relies on symmetry-protected edge states supported by interfaces between domains characterized by distinct topological invariants^2^. The synergy between non-Hermiticity and topology in active optical systems offers both new physics governed by non-Hermitian Hamiltonians^3,4^ and potential applications for the design of topological lasers with superior characteristics and tolerance to fabrication imperfections^5–11^. Recent experimental demonstrations include single-mode lasing based on a topologically protected edge state in micro-resonator arrays^8^ and unidirectional outcoupling of laser emission from magnetic-field biased photonic crystal cavities of arbitrary shapes^6^. Above the lasing threshold, it also becomes crucial to account for gain saturation and other nonlinear effects, such as Kerr self-action, that pose interesting new challenges in bridging topological photonics with nonlinear optics^12^.

Motivated by optical on-chip applications, special efforts have been made toward bringing topological photonics to the nanoscale^13–18^. Nanostructures made of high-index dielectric materials with judiciously designed resonant elements and lattice arrangements show special promise for the practical implementation of topological order for light. High-index dielectrics such as III–V semiconductors can be designed to contain strong optical gain further enhanced by topological field localization, forming a favourable platform for active topological nanophotonics^10,11,19^. In this direction, two recent pioneering studies employed photonic structures emulating a one-dimensional (1D) Su–Schrieffer–Heeger model^10,11^. Ota et al.^10^ reported single-mode lasing from topological defect modes created in a patterned nanobeam GaAs photonic crystal with embedded InAs quantum dots. Later, in a similar fashion, Han et al.^11^ demonstrated lasing driven by midgap edge states in a dimer chain of nanocavities arranged in a hexagonal InAsP/InP multiple-quantum-well photonic crystal. Most recently, a band-inversion mechanism was used to implement lasing in a bulk state trapped by a two-dimensional (2D) hexagonal cavity in a Kekulé-patterned InGaAsP slab^19^. The general exciting motivation of those studies is that the concepts of topology may serve as a significant guiding scheme for the smart control of the number, spectral separation, localization scales and quality factors of edge and defect modes in topological cavities for lasing. Pulsed optical pumping at powers relatively close to the lasing threshold assumes that observations can be largely explained in terms of the physics of linear modes. Harnessing topological phases and hyperuniformity^20^ in photonic nanostructures opens new horizons in designing advanced integrated nanoscale light sources with controllable properties.

Here, we study lasing from valley-Hall nanophotonic cavities embedded into 2D topological lattices^15,16,21^. The structures include III–V semiconductor quantum wells that act as a gain medium and serve as an internal light probe in the near infrared region. We demonstrate low-threshold lasing under uniform pumping from high-quality cavity modes hosted within the topological bandgap of the structure. While lasing in the first nanoscale topological systems has been associated with cryogenic temperatures^5,6,10^, we reach the lasing regime at room temperature.

## Results

### Valley-Hall topological cavities

**Fig. 1:**
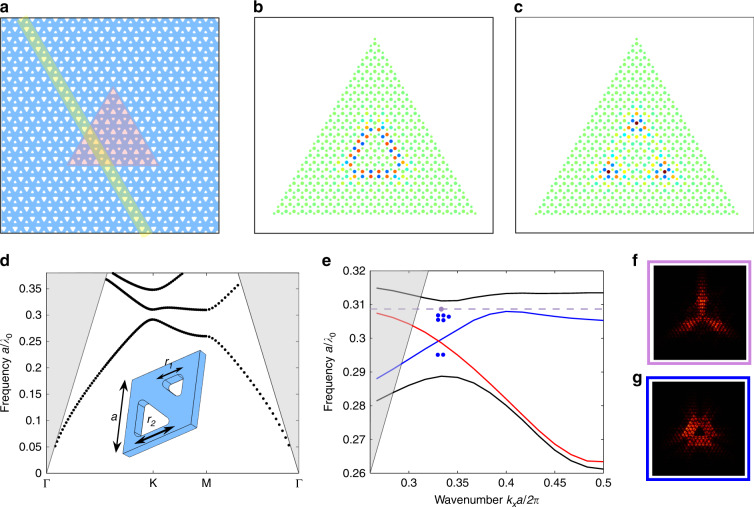
Valley-Hall topological photonic cavity. **a** A closed domain wall is created by inversion symmetry breaking in the dimerized graphene lattice and shaped in the form of an equilateral triangle (reddish area). The structure is implemented in a dielectric photonic crystal slab of a finite thickness. The shaded yellow stripe illustrates a supercell with a zigzag cut. **b**, **c** Tight-binding model visualization of optical modes of the structure. **d** Numerically calculated bulk photonic band structure for TE-like modes. The grey shaded regions indicate the light cone. The inset shows the unit cell and dimensions of the structure: lattice period *a* = 460 nm, sizes of triangulated holes *r*_1 _= 174 nm, *r*_2 _= 269 nm, and slab thickness fixed to 250 nm. A degeneracy is lifted, and the bandgap opens by dimerization between the first and second bands in the vicinity of the K point. **e** Projected bulk bands (black curves), spectra of edge states (red and blue lines) and cavity modes (dots). The dashed level marks the triade mode frequency. Characteristic distributions of the near-field intensity |**E**|^2^ in the (**f**) triade and (**g**) edge cavity modes at resonant wavelengths of 1.49 µm and 1.51 µm, respectively. Their simulated quality factors are **f** 4.3 × 10^3^ and **g** 1.9 × 10^3^

We design a topological cavity based on the closed valley-Hall domain wall created by the inversion of a staggered sublattice potential in a honeycomb periodic photonic lattice^15,16,21–25^. The triangle-shaped cavity is implemented in a dielectric membrane, as shown in Fig. 1a. Two triangular holes in each unit cell of the bipartite lattice have different sizes that leads to the opening of bandgaps near the Dirac points of the corresponding Brillouin zone.

The topological properties of the valley-Hall photonic lattices can be captured by employing the 2D tight-binding model^26,27^. In the second-quantization notations, the Hamiltonian can be written in the form:

1 $$\hat H = - \mathop {\sum}\nolimits_{ < i,j > } {t\hat a_j^\dagger \hat a_i} - {M} \mathop {\sum}\nolimits_i {( - 1)^i\hat a_i\hat a_i^\dagger }$$

where $$\hat a_i$$$$(\hat a_{{i}}^\dagger )$$ denote the creation (annihilation) operators in the *i*th unit cell, 〈*i*,*j*〉 indicate summations over the nearest neighbours sites, *t* > 0 is the hopping amplitude, and *M* is a parameter that breaks the inversion symmetry via sublattice detuning. In an application to the studied metasurface, the 2D tight-binding model can be viewed as coupled equations formulated for out-of-plane magnetic dipoles *m*_*z*_ with nearest-neighbour electromagnetic coupling. The field is predominantly TE polarized, and excitation around each hole has a dominant *H*_*z*_ component. Accordingly, a negative sign in front of the coupling constant *t* corresponds to the transverse dipole–dipole interaction.

The chiral edge states with linear dispersion traversing the bandgap can be inferred in the continuum limit from the massive Dirac Hamiltonian, applicable at |*t*|≫|*M*| near the corners of the hexagonal Brillouin zone (see Supplementary information, Sections I and II):

2 $$\hat H_D = v_D\left( {\hat \sigma _x\hat \tau _z\delta k_x + \hat \sigma _y\hat \tau _0\delta k_y} \right) - \hat \sigma _z\hat \tau _0M$$

Here, the Pauli matrices $\hat \sigma$ and $\hat \tau$ act on the sublattice and valley degrees of freedom, respectively; $v_D = \frac{{\sqrt 3 }}{2}ta$ is the velocity parameter; *a* is a lattice period; and *M* can be treated as an effective mass. This form of the effective electromagnetic Hamiltonian (2) can also be obtained for the 2D photonic crystal directly from Maxwell’s equations by the plane wave expansion method (see Supplementary information, Section III). For our structure, we assume *M* > 0. When *M* = 0, Eq. (2) describes the conical intersections in pristine graphene with a linear dispersion relation $\omega _ \pm = \pm v_D|\delta \bf k|$ resembling that of massless fermions. The degeneracies are protected by time-reversal TR and parity P (spatial inversion) symmetries. Breaking parity P (inversion) symmetry by adding a staggered sublattice potential lifts the degeneracies and opens a bandgap. The dispersion relations for two bulk bands are given by the expression $\omega _ \pm (\delta {\mathbf{k}}) = \pm \sqrt {v_D^2(\delta k_x^2 + \delta k_y^2) + M^2}$. Because the Berry curvatures at the *K*_±_ points, $K_ \pm = \frac{{4\pi }}{{3a}}( \pm 1,0)$, have opposite signs for this symmetry reduction, the Chern number vanishes. Nevertheless, the domain walls separating valley-Hall lattices that have opposite P breaking support chiral edge states. For small P breaking, the Berry curvatures:

3 $${\mathbf{F}}_ \pm = \mp \tau _z\frac{{v_D^2M}}{{2(M^2 + v_D^2\delta k^2)^{3/2}}}{\mathbf{z}}_{\mathrm{0}}$$

with $\tau _z(K_ \pm ) = \pm 1$ being the indices for the two valleys, are strongly localized. The local integrals $\gamma _ \pm = {\iint} {{\mathbf{F}}_ \pm \text{d}^2{\mathbf{k}}}$ computed in the reciprocal space over the area around *K*_±_ valleys (the Berry phase) take nonzero quantized values of ±*π* for each band, resulting in the valley Chern number $C_ \pm ^{{\mathrm{valley}}} = \frac{{\gamma _ \pm }}{{2\pi }} = \pm \tau _z\frac{1}{2}{\mathrm{sgn}}(M)$. Flipping the sign of the P breaking also flips the sign of the Berry curvature in each valley. Across a domain wall, there is a difference of ±1 between valley Chern numbers, which in accord with the general principle of bulk-boundary correspondence results in one family of topological edge states in each valley.

To obtain the edge waves localized at the domain walls separating two media with opposite masses, we look for the solutions bound to the interface $[\psi _1,\psi _2] \propto {\mathrm{exp}}(i\delta k_xx - {\it{\varkappa }}|y|)$ and use the parity dictated condition for the components of the wave-function column-vector $\psi _1(0) = \pm \psi _2(0)$ (a symmetric/antisymmetric spinor) (see details in Supplementary information, Section II.A). In this case, the effective Hamiltonian Eq. (2) yields the linear dispersion $\omega _{{\mathrm{e}}.{\mathrm{s}}.} = \mp v_D\delta k_x$ (opposite signs in different valleys imply opposite group velocities) and the constant decay factor ${\it{\varkappa }} = |M|/v_D$ of the valley edge states in the vicinity of the Dirac points. Importantly, these valley edge states are associated with the valley-Hall effect, and they can be gaped from bulk states^26^ by increasing the magnitude of *M*, as we discuss below. The counter-propagating valley-polarized edge states can be selectively excited by the point sources of opposite handedness placed near the interface. Topological cavities can be created by mass inversion in a circular domain (see Supplementary information, Section II.B). For cavities of radius ${\cal{R}}$ much larger than the decay length of the states into the bulk insulator, we obtain the cavity’s spectrum quantized by the integer azimuthal number $m:\omega (m) \approx - \frac{{v_D}}{{\cal{R}}}\left( {\frac{1}{2} \pm m} \right)$. It comprises edge states that occur in pairs with opposite pseudospins (here, valley acts as a pseudospin).

Alternatively, the edge states at the P-symmetric domain walls between dimerized crystals can be derived directly from the tight-binding model (1) (see Supplementary information, Section I.B) with a nonperturbative analytical method. For a zigzag cut, we obtain the dispersion relation of edge states in the form:

4 $$\omega _{{\mathrm{e}}.{\mathrm{s}}.} = \pm t - \sqrt {M^2 + 4t^2{\mathrm{cos}}^2(k_xa/2)}$$

At $t \lesssim 2M$, the spectrum contains a minigap between the edge state dispersion branches and projected bulk bands, which accommodates a corner mode. In the limit of weak coupling at small *t*≪*M*, the defect state appears at a trimer corner (composed of three equipotential sites), and its frequency emanates from the asymptote:

5 $$\omega _{{\mathrm{c}}.{\mathrm{s}}.} \approx - M + \sqrt 2 t - \frac{3}{4}\frac{{t^2}}{M}$$

With increasing *t*, the frequency of the corner mode gradually moves across the bandgap from −*M* to *M* and finally disappears, merging into the upper bulk band (see Supplementary information, Section I.C). Depending on the detuning Δ*t* from the critical value t*~2*M*, the frequency changes approximately as $\omega _{{\mathrm{c}}.{\mathrm{s}}.} \approx M\left( {1 - \frac{{3\Delta {\mathrm{t}}}}{{4M}} - \frac{{\Delta t^2}}{{4M^2}}} \right)$, while the localization scale rapidly decreases $\Lambda \sim a\sqrt {\frac{{2M}}{{\Delta t}}}$. The typical profiles of edge and corner modes calculated with the tight-binding model on a finite lattice are illustrated in Fig. 1b, c.

The numerically computed band structure for our slab metasurface with symmetry breaking is shown in Fig. 1d. First-principles simulations are performed for the InGaAsP slab with a fixed thickness of 250 nm, lattice periods around *a* = 460 nm, and side dimensions of triangulated holes near *r*_1_ = 0.6*a*, *r*_2_ = 0.4*a*. While the valley-Hall interface guides valley-polarized edge states, the designed small cavity supports a quantized spectrum of confined modes within the bandgap, eight modes in total, see Fig. 1e. We find these results in compliance with the tight-binding calculations using the effective parameters estimated by fitting the simulated band structure (see Supplementary information, Section I.C). Some distinctions between the cavity-mode profiles attained with the full-wave (see Fig. 1f, g) and tight-binding (see Fig. 1b, c) modelling can be associated with a finite thickness of the dielectric slab fostering leakage and long-range electromagnetic interactions beyond the nearest neighbours. The red and blue curves of the edge state dispersion in Fig. 1e correspond to two possible configurations of the zigzag domain wall—with smaller and larger holes near the interface, respectively. Blue dots depict the discrete spectrum of the cavity illustrated in Fig. 1a. Among the gap states, as a candidate for lasing, we specifically distinguish the triade cavity mode, which confines light at the three corners in-phase and exhibits a high estimated quality factor of about 4000. In its genesis, this mode is different from the corner states in quadrupolar topological insulators^28–30^ and distorted photonic lattices^31–33^. Despite the complexity of a real electromagnetic structure, the prerequisite of this bound-state formation at the valley-Hall domain wall is adequately captured within the nearest-neighbour tight-binding approximation. While the midgap behaviour in this system is governed by the Dirac-like Hamiltonian in two valleys, the deviation from the linear dispersion relation of the edge states closer to the upper band facilitates field localization at the corner defect. The qualitative description of such trapping is outlined in the Supplementary information, Section II.C. This specific topological defect is formed at the intersection of two valley-Hall domain walls. Because the chiral symmetry is broken, the frequency of the corner mode is not pinned, and the variations in the parameters (*t*/*M* in the tight-binding approach) shift the bound-state frequency within the gap. However, this corner mode survives disorder that is weak compared to the gap size and shows a desirable confinement provided perturbations in the corner area do not destroy the minigap. For instance, we obtain numerically that when increasing the triangle side length from 7 to 9 lattice periods, the real part of the triade mode frequency changes only slightly by 0.3 THz. If we introduce an edge defect, such as a missing hole, by two unit cells close to the corner, then the mode also remains well defined with the modal profile locally perturbed.

### Optical characterization

**Fig. 2:**
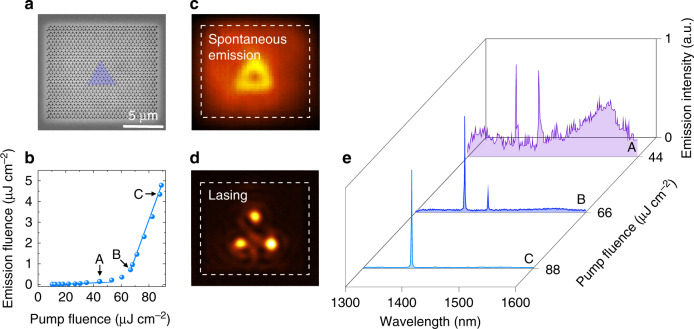
Lasing in topological cavities. **a** Scanning electron microscope image of the fabricated sample. The false-colour triangle marks the interior of the topological cavity. **b** Emission energy vs. the pump energy dependence showing a threshold transition to lasing. The sample is optically pumped at a 980 nm wavelength with 8 ns pulses at a 10 kHz repetition rate. Spatial distribution of emission for the pump intensity **c** below and **d** above the lasing threshold. **e** Emission spectra showing a transition to a narrow-linewidth lasing. In the lasing regime, the FWHM (full width at half maximum) does not exceed 2 nm and is resolution limited by the spectrometer

We fabricate topological cavities from a 250 nm-thick InGaAsP slab that incorporates three quantum wells using an electron-beam lithography and dry-etching process (see SEM image in Fig. 2a). The fabrication parameters are close to those used in the theoretical calculations. At the domain wall, two larger holes are positioned facing each other. The length of one side of our triangular topological cavity is 7 lattice periods, ~3.22 µm.

Next, we optically pump the sample using 8 ns FWHM laser pulses with 980 nm wavelength and 10 kHz repetition rate. The spot size of a pump laser is comparable to the full sample size. We studied the emission intensity vs. the pump intensity. The light in – light out curve in Fig. 2b exhibits a clear lasing threshold, which is the characteristic lasing behaviour. The lasing saturates at higher pump intensities. Spatial distributions of light emission below and above the threshold are depicted in Fig. 2c, d, respectively. The field profile in Fig. 2c shows the enhancement along the entire topological domain wall associated with edge states. The mode profile in Fig. 2d shows intensity confinements at the three corners. This triade mode is a topological cavity mode that facilitates lasing due to its high-quality factor and small mode volume. We further perform spectral measurements of the emission (see Fig. 2e) at three levels of pump intensity. The purple curve is measured below the lasing threshold and corresponds to the spatial distribution of the emission depicted in Fig. 2c. The spectrum features two maxima, which we attribute to the triade and edge modes residing within the topological bandgap. The light-blue curve is measured above the lasing threshold, and it exhibits the single-mode operation with the spatial profile of the emission depicted in Fig. 2d.

**Fig. 3:**
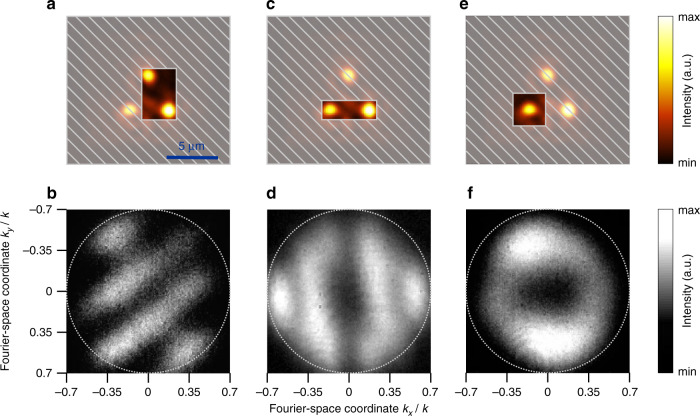
Coherence and far-field patterns of the laser emission **a–d** Experimental study of the coherence of the emission observed in Fig. 2d. **a**, **c** When two emission spots are isolated, **b**, **d** interference fringes are observed in Fourier space. **e**, **f** Real space emission distribution and Fourier-space emission directionality from an isolated cavity corner. An experimentally observed donut-like directionality pattern suggests an optical singularity hosted by the laser beam

We experimentally examine the coherence of the emission by studying its far-field directionality pattern (see “Materials and methods”). When two emission spots are isolated in the real space, as in Fig. 3a, c, we observe an interference pattern in the Fourier space (see Fig. 3b, d), suggesting that the emission is coherent. With these experimental evidences, we conclude that three corner modes are coupled to each other and exhibit lasing action.

After submission of this paper, we became aware of related theoretical studies of active honeycomb waveguide arrays^34^ and Kagome-patterned semiconductor slabs^35^. The most recent experimental efforts demonstrated an electrically pumped terahertz quantum cascade laser using the topological edge states of a valley-Hall photonic crystal^36^. However, none of those works reported triade mode lasing at the nanoscale.

**Fig. 4:**
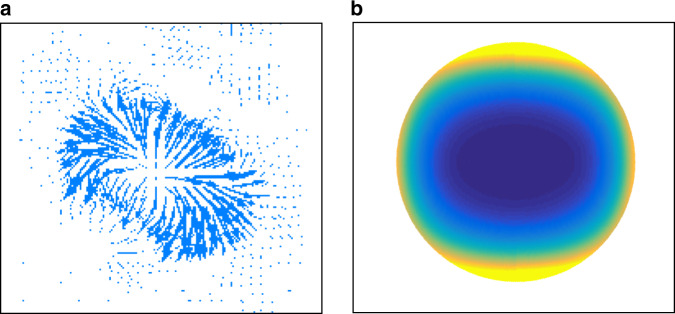
Singular emission from the domain wall corner. **a** Distribution of the Poynting vector, plotted as arrows around an isolated lasing cavity corner, indicates a singularity point. Numerically calculated with the 3D finite-element method for the triade eigenmode depicted in Fig. 1f. **b** Modelled far-field intensity pattern of the top corner

Finally, we isolate an individual lasing spot and measure its directionality diagram in Fourier space (Fig. 3f). We observe a donut-shaped beam carrying a singularity. We note that the singularity is observed only in Fourier space, while the real space image (Fig. 3e) shows the position of the emitter, not the directions of emanating light. The spatial structure of the emitted beam is defined by the mode profile, which can be modelled by a certain distribution of magnetic dipoles near the corner. The numerically retrieved distribution of the Poynting flux is shown in Fig. 4a. Based on the simulated far-field diagram in Fig. 4b, we identify the topological charge of +1. This singularity index is found from the line pattern of the far-field polarization around the dark direction. The selective excitation of the corners can be implemented via directional evanescent coupling with a spin-polarized source^18^. In this way, the directional coupling stems from the chirality of the evanescent edge states excited by the source operating at frequencies within the minigap. As a result, sources of opposite handedness evanescently couple to and excite the opposite corners of the cavity (see Supplementary Fig. S5). In turn, individual corner modes emit singular beams in the far field, as demonstrated in Figs. 3f and 4b.

## Discussion

We have realized active topological cavities created in 2D valley-Hall photonic structures. The active elements of the cavities are provided by embedded layers of III–V semiconductor quantum wells that act as a gain medium. We have demonstrated room-temperature lasing with these 2D nanoscale topological cavities. The cavity modes were probed and visualized by means of photoluminescence measurements. Above a certain pumping threshold, lasing occurs from a single tightly confined gap state. The corner field localization in a triade cavity mode gives rise to a donut-shaped lasing emission hosting a singularity. The proposed all-dielectric platform holds promise for the versatile design of active topological metasurfaces with integrated light sources. We envision that nontrivial topology combined with an optical gain has vast potential for advances in photonic applications of low-power nanoscale lasers operating at ambient temperatures.

## Materials and methods

### Sample fabrication

A scheme of the fabrication procedure is outlined in Supplementary Fig. S7. The wafer consists of a 250-nm-thick InGaAsP/800-nm-thick InP/100-nm-thick InGaAs/InP substrate. The InGaAsP slab includes three quantum wells with a central emission wavelength of 1.5 µm. Electron-beam lithography is carried out to define a PMMA mask on the InGaAsP slab, and chemically assisted ion-beam etching is performed to make holes on the slab. The PMMA layer is removed by using an oxygen plasma. Then, the free-standing slab structure is formed by wet-etching the sacrificial InP layer underneath the InGaAsP slab using a diluted HCl:H_2_O (3:1) solution at room temperature.

### Analytical and numerical approaches

To derive the effective Hamiltonian and topological properties of the valley-Hall photonic crystal, we use the tight-binding approach as well as the plane wave expansion method (see Supplementary information, Sections I and III). The full-wave numerical modelling of the slab metasurface is performed with a finite-element method solver COMSOL Multiphysics. The refractive index of the slab is set constant at 3.17 over the whole spectral range of interest. To calculate the photonic bulk and edge band structures (see Fig. 1e), we carry out eigenfrequency analyses in 3D setting for a bipartite (hexagonal or rhomboid) unit cell and a ribbon-shaped supercell, which is composed of stacking 20 unit cells and includes two domain walls. The perfectly matched layer and Floquet periodic boundary conditions are imposed in the vertical and lateral directions, respectively. The optical modes of the triangular topological cavity (see Fig. 1f, g) are simulated numerically by using the 3D eigenmode solver. For the triade mode depicted in Fig. 1f, we cut an area of about 1.5 lattice periods in the vicinity of the top corner. The emitting source from this area is then modelled as a distribution of magnetic dipoles approximately matching the *H*_*z*_ component distribution in the metasurface. Their interference leads to a singularity in the out-of-plane direction, as shown in the calculated radiation diagram of Fig. 4b.

### Experimental system

We pump the sample with a pulsed laser Femtolux Ekspla. We narrow down the beam size with a *f* = 300 mm lens and an objective lens Mitutoyo Plan Apo NIR HR (X100, 0.7 NA) such that the excitation area is approximately twice as large as the sample size. We build an image of the sample in reflection on an IR camera Xenics Bobcat 320 with an infinity-corrected achromatic lens *f* = 150 mm. In reflection, we block the pump beam with a 1300 nm long-pass filter. We measure the spectrum of the emission with an Ocean Optics NIR-Quest fibre-coupled spectrometer. The emission intensity vs. pump intensity is measured with an Ophir power metre.

For Fourier-space imaging, we introduce two additional lenses and a four-edge field diaphragm. The first lens with *f* = 75 mm is confocal with the objective back focal plane. The field diaphragm is placed in the back focal plane of the first lens; thus, in the image plane of the sample, real space filtering is performed. The second lens *f* = 50 mm is removable, and it is confocal with the first lens. When the second lens is present, the camera images the real space. When the second lens is absent, the camera images the Fourier space, that is the far-field directionality diagram of the emission. Fourier-space imaging is performed with an optical pump at a 1030 nm wavelength, with 2 ps pulses and 4 MHz repetition rate.

## Supplementary information

Supplementary Information
