# Supplementary material for: Room-temperature lasing from nanophotonic topological cavities
Source: Light Sci Appl. 2020 Jul 20;9:127. doi: 10.1038/s41377-020-00350-3 (PMC7371636; doi:10.1038/s41377-020-00350-3)
Supplement: Supplementary file 1 — Supplementary Information [file 41377_2020_350_MOESM1_ESM.pdf]

Supplementary Information for "Room-temperature lasing from nanophotonic topological cavities"

Daria Smirnova[1,2,*], Aditya Tripathi[1,3,*], Sergey Kruk[1,*], Min-Soo Hwang[4,*], Ha-Reem Kim[4], Hong-Gyu Park[4], and Yuri Kivshar[1]

[1] *Nonlinear Physics Center, Research School of Physics, Australian National University, Canberra ACT 2601 Australia*

[2] *Institute of Applied Physics, Russian Academy of Science, Nizhny Novgorod 603950, Russia*

[3] *Department of Physics, Indian Institute of Technology, Delhi 110016, India*

[4] *Department of Physics, Korea University, Seoul 02841, Republic of Korea*

[*] These authors contributed equally to the present work

I. Tight-binding model of staggered graphene

A. Derivation of the low-energy Effective Hamiltonian

We consider a graphene lattice which represents a triangular lattice with two elements per unit cell and thereby it consists of two sublattices A and B. We denote the spacing between neighboring sites by a_0. Two basis vectors of the Bravais lattice are $\mathbf{a}_1 = a(1,0)$, $\mathbf{a}_2 = a(-1/2, \sqrt{3}/2)$, where $a = \sqrt{3}a_0$ is a lattice period. The primitive translation vectors of the reciprocal lattice are then found from the condition $(\mathbf{b}_i, \mathbf{a}_j) = 2\pi\delta_{ij}$: $\mathbf{b}_1 = G\,(\sqrt{3}/2, 1/2)$, $\mathbf{b}_2 = G\,(0,1)$, where $G = 4\,\pi/(\sqrt{3}\,a)$. The Brillouin zone is a hexagon with two inequivalent corners $\mathbf{K}_\pm = \dfrac{4\pi}{3a}(\pm 1, 0) \equiv K(\pm 1, 0)$. The in-plane vector $\mathbf{K}_\pm$ is unchanged by the rotation operation $C(\mathbf{z}_0, 2\pi/3) = C_3$ with respect to the center of the hexagon. An element on sublattice A has three neighboring elements, all on sublattice B, at displacements $\boldsymbol{\delta}_1 = (0, a_0)$, $\boldsymbol{\delta}_2 = (\sqrt{3}a_0/2, -a_0/2)$, $\boldsymbol{\delta}_3 = (-\sqrt{3}a_0/2, -a_0/2)$. An element on sublattice B has three neighbors on sublattice A at displacements $-\boldsymbol{\delta}_1$, $-\boldsymbol{\delta}_3$, $-\boldsymbol{\delta}_2$. We look for the solutions in the form $\Psi_{A,B} = \psi_{1,2} e^{-i\omega t + i\mathbf{k}\mathbf{r}}$ on sublattices A and B. Assuming the coupling between the adjacent resonant elements of artificial optical graphenes can be treated similar to electron hopping in their electronic counterparts, the tight-binding approximation can be adopted. Based on the tight-binding model for dynamics $i\partial_t \Psi = \hat{H}\Psi$ in a honeycomb lattice, the amplitudes $\psi_{1,2}$ and eigenvalue ω are determined from two equations

$$-t\left(e^{i\mathbf{k}\boldsymbol{\delta}_1} + e^{i\mathbf{k}\boldsymbol{\delta}_2} + e^{i\mathbf{k}\boldsymbol{\delta}_3}\right)\psi_2 = (\omega - \omega_A)\psi_1 \tag{S1a}$$

$$-t\left(e^{-i\mathbf{k}\boldsymbol{\delta}_1} + e^{-i\mathbf{k}\boldsymbol{\delta}_2} + e^{-i\mathbf{k}\boldsymbol{\delta}_3}\right)\psi_1 = (\omega - \omega_B)\psi_2\,, \tag{S1b}$$

or equivalently,

$$-t\left(e^{ik_y a_0} + 2e^{-ik_y a_0/2}\cos\frac{\sqrt{3}}{2}k_x a_0\right)\psi_2 = \omega\psi_1 \tag{S2a}$$

$$-t\left(e^{-ik_y a_0} + 2e^{ik_y a_0/2}\cos\frac{\sqrt{3}}{2}k_x a_0\right)\psi_1 = \omega\psi_2\,, \tag{S2b}$$

where t is the tunneling constant and $\omega_{A,B}$ are eigenfrequencies. Solving the eigenvalue problem, we get dispersion of bulk modes

$$\omega_\pm = \omega_0 \pm \sqrt{M^2 + t^2(1 + 4\cos^2(k_x a/2) + 4\cos(k_x a/2)\cos(k_y a\sqrt{3}/2))}\,, \tag{S3}$$

where $\omega_0 = (\omega_A + \omega_B)/2$ is the midgap frequency, and $M = (\omega_A - \omega_B)/2$ is the effective mass. Dimerization opens the band gap $((-|M|, |M|)$ in two valleys in the vicinity of $K_\pm$ points. Near the Dirac points ($k_x = \pm K + \delta k_x, \delta k_y = 0$), Eq. (S3) recasts to

$$\omega_\pm = \omega_0 \pm \sqrt{M^2 + \frac{3}{4}t^2 a^2 \delta k_x^2}\,. \tag{S4}$$

Without loss of generality, we may set the midgap level $\omega_0 = 0$ and assume ω is a deviation from the midgap frequency.

Equations (S2) can be treated as a matrix equation $\omega[\psi_1, \psi_2] = \hat{H}(\mathbf{k})[\psi_1, \psi_2]$, where $\hat{H}(\mathbf{k})$ conforms to the two-band model of the general form,

$$\hat{H}(\mathbf{k}) = \begin{pmatrix} h_z & h_{||} \\ h_{||}^* & -h_z \end{pmatrix} = \begin{pmatrix} h_z & h_x - ih_y \\ h_x + ih_y & -h_z \end{pmatrix} = \mathbf{h}(\mathbf{k}) \cdot \hat{\sigma} \tag{S5}$$

where $\hat{\sigma} = (\sigma_x, \sigma_y, \sigma_z)$ is a vector consisting of the three Pauli matrices, and the vector $\mathbf{h}(\mathbf{k}) = (h_x(\mathbf{k}), h_y(\mathbf{k}), h_z(\mathbf{k}))$ captures topology and provides a convenient way for its visualization. The vector $\mathbf{h}(\mathbf{k})$ determines pseudo-spin textures for the eigenstates of the model. Specifically for staggered graphene, we have $h_{||}(k_x, k_y) = -t\left(e^{ik_y a/\sqrt{3}} + 2e^{-ik_y a/2\sqrt{3}} \cos(k_x a/2)\right)$, $h_z = M$.

Expanding $h(\mathbf{k})$ in the vicinity of the Dirac points $\mathbf{k} = \mathbf{K}_\pm + \delta\mathbf{k}$, where $\delta\mathbf{k}$ is a small detuning, we obtain the effective Hamiltonian at two valleys

$$\hat{H}_{K_\pm}(\delta\mathbf{k}) = \begin{pmatrix} M & v_F(\pm\delta k_x - i\delta k_y) - \mu(\delta k_x \pm i\delta k_y)^2 \\ v_F(\pm\delta k_x + i\delta k_y) - \mu(\delta k_x \mp i\delta k_y)^2 & -M \end{pmatrix}, \tag{S6}$$

where $v_D = \frac{\sqrt{3}}{2}ta$ is the velocity parameter, and $\mu = \frac{ta^2}{8}$. Keeping only the first order in $\delta\mathbf{k}$, we get

$$\hat{H}_{K_\pm}(\delta\mathbf{k}) = M\hat{\sigma}_z \pm v_D \delta k_x \hat{\sigma}_x + v_D \delta k_y \hat{\sigma}_y , \tag{S7}$$

where $\delta U = [M, -M]$ is the staggered onsite potential.

B. Analytical solution for edge states at the domain wall

Next, we consider a honeycomb lattice with a staggered on-site potential in the stripe geometry. The stripe consists of two domains with the zigzag interface, where the sign of the parity breaking is flipped, as shown in Fig. S1(a). The tight-binding model for this lattice is written as a system of discrete equations:

$$\begin{cases} \omega\psi_{I,A}(n) = -t\psi_{I,B}(n+1) - 2t\cos(k\rho)\psi_{I,B}(n) + M\psi_{I,A}(n), & n = 0,1,2,...N-1, \\ \omega\psi_{I,B}(n) = -t\psi_{I,A}(n-1) - 2t\cos(k\rho)\psi_{I,A}(n) - M\psi_{I,B}(n), & n = 1,2,...N, \\ \omega\psi_{II,A}(n) = -t\psi_{II,B}(n-1) - 2t\cos(k\rho)\psi_{II,B}(n) - M\psi_{II,A}(n), & n = 1,2,...N, \\ \omega\psi_{II,B}(n) = -t\psi_{II,A}(n+1) - 2t\cos(k\rho)\psi_{II,A}(n) + M\psi_{II,B}(n), & n = 0,1,2,...N-1, \end{cases} \tag{S8}$$

where $k \equiv k_x$ is the momentum vector along the x-aligned interface, $\rho = a/2$, $\psi_{s,j}(n)$ is the field at site n, j located in the domain $s = I, II$, index $j = A, B$ denotes sublattice, and integer argument n is a dimer number. At the domain wall, the TBM equations are:

$$\begin{cases} \omega\psi_{I,B}(0) = -t\psi_{II,A}(0) - 2t\cos(k\rho)\psi_{I,A}(0) - M\psi_{I,B}(0) , \\ \omega\psi_{II,A}(0) = -t\psi_{I,B}(0) - 2t\cos(k\rho)\psi_{II,B}(0) - M\psi_{II,A}(0) . \end{cases}$$

The stripe is considered finite, which formally implies the following boundary conditions at the external boundaries of the stripe

$$\psi_{I/II,B/A}(N+1) = 0. \tag{S9}$$

Here, we focus on the edge states localized at the domain wall. They are concentrated at sites (0,B) in domain I and sites (0,A) in domain II. The solutions for the edge states assume the form:

$$\begin{aligned} \psi_{I,A}(n) &= a_I e^{-\varkappa(n+1)\frac{\sqrt{3}}{2}a}, \quad \psi_{I,B}(n) = b_I e^{-\varkappa n\frac{\sqrt{3}}{2}a} \\ \psi_{II,A}(n) &= a_{II} e^{-\varkappa n\frac{\sqrt{3}}{2}a}, \quad \psi_{II,B}(n) = b_{II} e^{-\varkappa(n+1)\frac{\sqrt{3}}{2}a} \end{aligned} \tag{S10}$$

The parameter $\varkappa^{-1}$ characterizes the decay length away from the interface. Due to parity symmetry of the system, the amplitudes at the interface are connected as

$$b_I = Ea_{II}, \tag{S11}$$

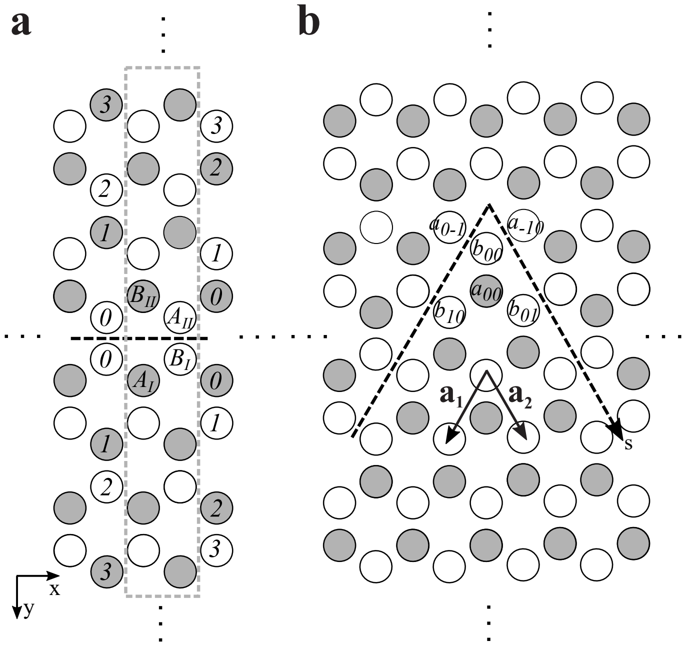

FIG. S1: Geometries of zigzag-cut interfaces: (a) straight x-aligned domain wall, (b) corner-shaped domain wall.

where the sign $E = \pm 1$ corresponds to symmetric/antisymmetric solutions with respect to the interface. Utilizing the ansatz (S10) with the boundary conditions, we get the continuity condition for the components $a_I = a_{II}$, $b_I = b_{II}$.

Substituting Eq. (S10) into Eq. (S8) (for domain I) results in a system of two equations

$$\begin{cases} \omega a_I = -tb_I - 2t\cos(k\rho) b_I e^{\varkappa \frac{\sqrt{3}}{2} a} + M a_I, \\ \omega b_I = -t a_I - 2t\cos(k\rho) a_I e^{-\varkappa \frac{\sqrt{3}}{2} a} - M b_I. \end{cases} \tag{S12}$$

We then apply the condition $b_I = E a_I$

$$\begin{cases} \omega = -tE - 2tE\cos(k\rho) e^{\varkappa \frac{\sqrt{3}}{2} a} + M, \\ \omega = -tE - 2tE\cos(k\rho) e^{-\varkappa \frac{\sqrt{3}}{2} a} - M. \end{cases}$$

and derive the dispersion relation of edge states in the form

$$\omega = -tE \pm \sqrt{M^2 + 4t^2\cos^2(k\rho)}, \tag{S13}$$

which can be rewritten as

$$\omega = \begin{cases} \pm t - \sqrt{M^2 + 4t^2\cos^2(k\rho)}, \\ \pm t + \sqrt{M^2 + 4t^2\cos^2(k\rho)}. \end{cases} \tag{S14}$$

Four valley edge states with different parities are found, among which two are localized at the interface made of a dimer of negative mass $-|M|$ and the other pair is confined to the positive mass $|M|$ dimer.

Near the Dirac point at $k_x = 4\pi/3a$, we can employ the expansion $\cos(k\rho) = \cos(k_x a/2) \approx -\frac{1}{2} - \frac{\sqrt{3}}{4}\delta k_x a$, and for the antisymmetric edge state the spectrum is simplified to

$$\omega \approx t - \sqrt{M^2 + t^2 + t^2\sqrt{3}\delta k_x a} \tag{S15}$$

We find out that Eq. (S15) for $|M| \ll |t|$ transforms to $\omega \approx -v_D \delta k_x$, while for $|t| \ll |M|$ it is approximated by $\omega \approx t - M\left[1 + t^2\left(\frac{1+\sqrt{3}\delta k_x a}{2M^2}\right)\right]$. The decay rate depends on the propagation constant as follows

$$\varkappa \frac{\sqrt{3}}{2} a = \ln\left(\frac{\sqrt{M^2 + 4t^2\cos^2 k\rho} + |M|}{|2t\cos k\rho|}\right). \tag{S16}$$

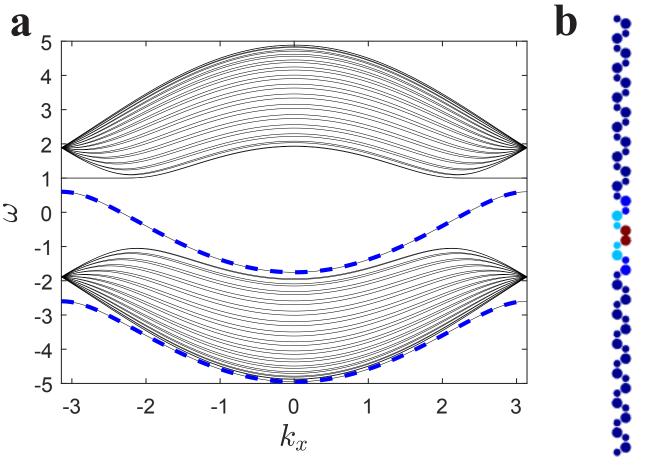

FIG. S2: (a) Numerically calculated spectrum (black color) of the finite zigzag graphene ribbon with a P-symmetric domain wall. Overlaid dashed blue curves are dispersion branches of edge states found analytically. (b) Profile of the valley-Hall edge state localized at the domain wall. TBM parameters $|M| = 1$, $t = 1.6$, $a = 1$.

At $|M| \ll |t|$ this expression turns to $\varkappa = |M|/v_D$, that suggests a constant decay factor for the valley edge states near the Dirac points.

Alternatively, spectrum of edge states can be calculated numerically by solving the eigenvalue problem for a zigzag ribbon composed of two domains. We first compose $N \times N$ matrix for one of the domains

$$\hat{H}_I = \begin{pmatrix} M & -2t\cos(k\rho) & 0 & 0 & \cdots & 0 \\ -2t\cos(k\rho) & -M & -t & 0 & \cdots & 0 \\ 0 & -t & M & -2t\cos(k\rho) & \cdots & 0 \\ \vdots & \vdots & \vdots & \vdots & \ddots & \vdots \\ 0 & 0 & 0 & \cdots & -2t\cos(k\rho) & -M \end{pmatrix} \tag{S17}$$

The second matrix $\hat{H}_{II}$ is obtained by replacement $M \to (-M)$. The final matrix $\hat{H}_{\rm F}$ for a finite stripe of size $2N \times 2N$ is then constructed by stitching the two matrices and incorporating a bond between them in the matrix elements $H_{\rm F}(N, N+1) = -t$, $H_{\rm F}(N+1, N) = -t$. The outer zigzag cut of such finite ribbon supports edge states with a flat band dispersion $\omega = M$. The analytically derived dispersion of the edge modes perfectly agrees with the numerical tight-binding calculations, see Fig. S2. Imposing periodic boundary conditions $H_{\rm F}(1, 2N) = -t$, $H_{\rm F}(2N, 1) = -t$ corresponds to the geometry with two domain walls, whose spectrum includes all 4 solutions given by Eq. (S14).

C. Corner states

In the limit of weak coupling $|t| \to 0$, $|t| \ll |M|$, we can find the frequencies of edge states from two coupled equations for a dimer $a_{II}(0), b_I(0)$ at the interface

$$\begin{pmatrix} -M-\omega & -t \\ -t & -M-\omega \end{pmatrix} \begin{pmatrix} a \\ b \end{pmatrix} = 0. \tag{S18}$$

As above, we assume $(-M)$ interface in the geometry of Fig. S1. Splitting of the level results in frequencies $\omega_{1,2} = -M \pm t$ with symmetric and antisymmetric eigenfunctions

$$\begin{pmatrix} a \\ b \end{pmatrix}_{1,2} = \begin{pmatrix} 1 \\ \mp 1 \end{pmatrix} . \tag{S19}$$

Similarly, in the limit $|t| \ll |M|$, for the states localized at one corner depicted in Fig. S1(b), we write approximate

solutions based on the coupled equations for three coupled sites (a corner trimer)

$$\begin{pmatrix} -M-\omega & -t & 0 \\ -t & -M-\omega & -t \\ 0 & -t & -M-\omega \end{pmatrix} \begin{pmatrix} a_1 \\ b \\ a_2 \end{pmatrix} = 0, \tag{S20}$$

which yields three levels $\omega_1 = -M$, $\omega_{2,3} = -M \pm \sqrt{2}t$, and corresponding eigenvectors:

$$\mathcal{V}_1 = \begin{pmatrix} a_1 \\ b \\ a_2 \end{pmatrix}_1 = \begin{pmatrix} 1 \\ 0 \\ -1 \end{pmatrix}, \quad \mathcal{V}_{2,3} = \begin{pmatrix} a_1 \\ b \\ a_2 \end{pmatrix}_{2,3} = \begin{pmatrix} \mp 1/\sqrt{2} \\ 1 \\ \mp 1/\sqrt{2} \end{pmatrix}. \tag{S21}$$

These simple considerations give us basic intuition. With increasing t, the frequency of the corner state gradually moves across the band gap from $-M$ to M, and finally disappears merging into the upper bulk band.

In the schematic Fig. S1, we distinguish two sublattices and dimers positioned at $(l\mathbf{a}_1 + h\mathbf{a}_2)$ by two integer Miller indices l, h. Here we choose the basis vectors $\mathbf{a}_1 = a(1/2, -\sqrt{3}/2)$, $\mathbf{a}_2 = a(-1/2, -\sqrt{3}/2)$. In the next order in $t/|M|$, we analytically derive

$$\omega_2(t \ll M) = -M + \sqrt{2}t - \frac{3}{4}\frac{t^2}{M} + \ldots, \tag{S22}$$

and the corresponding distribution of the excited elements near the corner

$$\begin{pmatrix} b_{00} \\ a_{-10} \\ a_{0-1} \\ a_{00} \\ b_{-10} \\ b_{0-1} \\ b_{-11} \\ b_{1-1} \end{pmatrix} \approx \begin{pmatrix} 1 \\ -\frac{1}{\sqrt{2}} + \frac{t}{8M} \\ -\frac{1}{\sqrt{2}} + \frac{t}{8M} \\ \frac{t}{2M} \\ -\frac{t}{2\sqrt{2}M} \\ -\frac{t}{2\sqrt{2}M} \\ -\frac{t}{2\sqrt{2}M} \\ -\frac{t}{2\sqrt{2}M} \end{pmatrix}. \tag{S23}$$

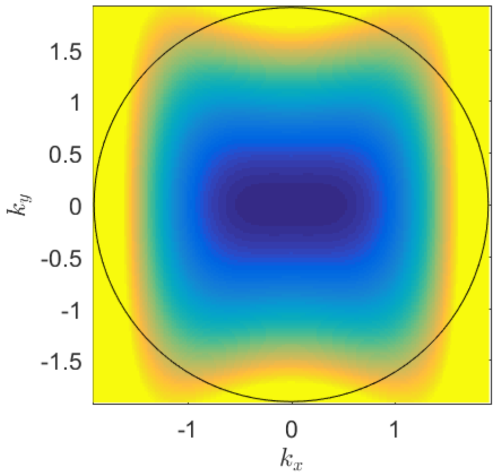

FIG. S3: Colormap of $|\tilde{V}(\mathbf{k})|^2$ distribution showing a singular point in the center (a solution of the scalar problem). Black circle corresponds to the free-space wavenumber magnitude.

When $t \approx 2M$, the frequency of the lowest-frequency staggered mode $\mathcal{V}_2$ is approaching $\omega \approx M$. Writing equation for three corner sites (for localized solutions the nearest neighbor a_{00} remains negligible, $|a_{00}| \ll |b_{00}|$),

$$(-\omega - M)b_{00} - t(a_{-10} + a_{0-1}) = 0\,, \tag{S24}$$

and taking into account a mirror symmetry of the structure, $a_{-10} = a_{0-1}$, we deduce $a_{-10} = a_{0-1} \approx -b_{00}/2$. Remarkably, these relations are found in agreement with the numerically calculated distribution of the out-of-plane magnetic field component H_z near the domain-wall corner in the triade cavity mode of our open metasurface. The field is seen primarily concentrated around these three holes. Next, for sake of simplicity, we assume that a reduced scattering potential $V(\mathbf{r})$ associated with the corner mode is dominated by this distribution and can be roughly described as delta functions with a weight V_0 centered at the air holes:

$$V(\mathbf{r}) = V_0 \left(\delta(\mathbf{r} + \boldsymbol{\delta}_1) - \frac{1}{2}\delta(\mathbf{r} - \boldsymbol{\delta}_2) - \frac{1}{2}\delta(\mathbf{r} - \boldsymbol{\delta}_3) \right)\,, \tag{S25}$$

where $\boldsymbol{\delta}_n$ are offsets of elements with respect to the corner hexagon center. The Fourier transform $\tilde{V}(\mathbf{k})$ is obtained by integrating the potential

$$\tilde{V}(\mathbf{k}) = \int V(x,y)\, e^{-i\mathbf{k}\cdot\mathbf{r}}\, d^2\mathbf{r} = V_0 e^{ik_y \frac{a_0}{2}} \left(\cos\left(k_y \frac{a_0}{2}\right) - \cos\left(k_x \frac{\sqrt{3}a_0}{2}\right) + i \sin\left(k_y \frac{a_0}{2}\right) \right)\,. \tag{S26}$$

Being multiplied by the radiation pattern of the elements (simplistically treated as point magnetic dipoles), it essentially determines the far-field diagram in the reciprocal space. The norm of this transform given by

$$|\tilde{V}(\mathbf{k})|^2 = V_0^2 \left(1 - 2\cos\left(\frac{k_y a_0}{2}\right) \cos\left(\frac{\sqrt{3}k_x a_0}{2}\right) + \cos^2\left(\frac{\sqrt{3}k_x a_0}{2}\right) \right) \tag{S27}$$

hosts a zero-order singularity and exhibits elliptical deformation, as shown in Fig. S3 calculated for approximate parameters of the structure studied experimentally.

Fitting numerically calculated band structures of the photonic metasurface (with varying structural parameters as described in the main text), we estimate effective parameters of the tight-binding model, $t/M \sim 1.8 - 2.2$, $a = 1$. Figure S4 shows an exemplary spectrum of a small-scale triangular cavity depicted in Fig. 1(b,c) of the main text. In the tight-binding calculations, the cavity is embedded in a large rectangular domain with a side length of 30 dimers. The band gap contains 8 modes, the triade cavity mode composed of coupled in-phase corner states is marked with a red circle.

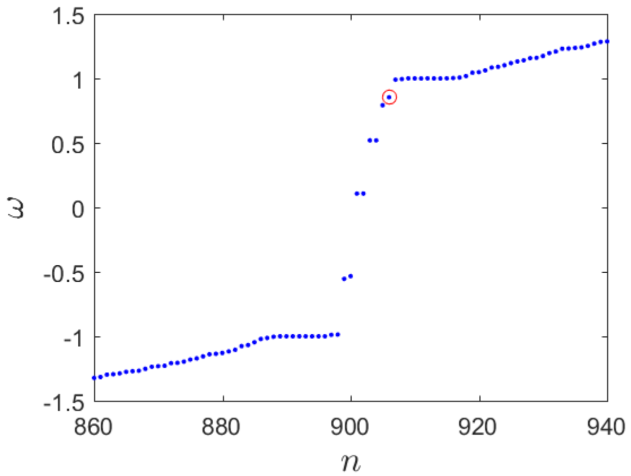

FIG. S4: Discrete spectrum of a triangular cavity. Frequency is plotted vs eigenmode number. Shown is a zoomed frame of the band gap. TBM parameters: $M = 1$, $t \sim 2M$, $a = 1$. The circled frequency corresponds to the triade cavity mode.

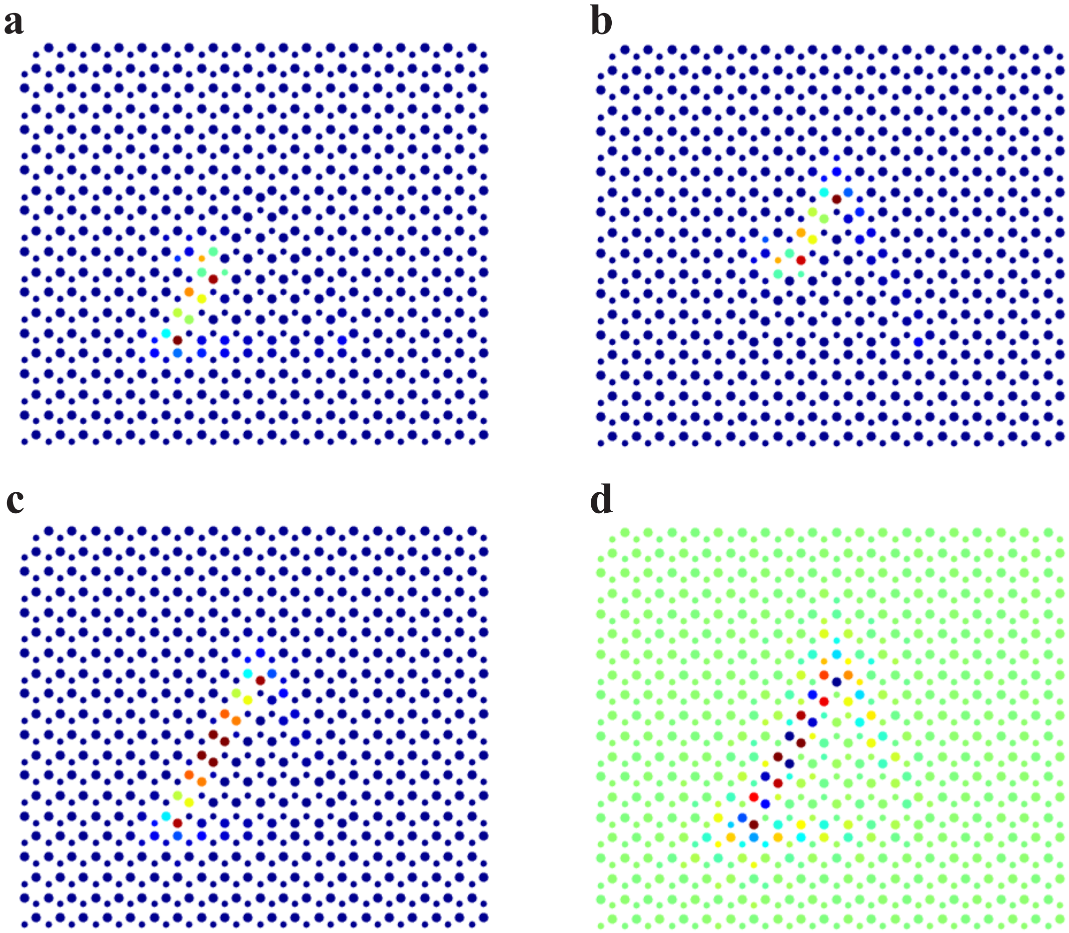

FIG. S5: (a,b) Snapshots of the field intensity distributions excited by the circularly polarized sources of opposite handnessness (calculated in the evolution dynamics). The field distribution trapped by the corner corresponds to the corner mode profile. (c,d) Stationary solution driven by the source evanescently coupled to two opposite corners: (c) intensity, (d) relative phase. The top and left bottom corners are excited in the opposite phase. Sources are located in the midside of a triangle. TBM parameters: $M = 1$, $t = 1.5M$, $a = 1$, $\omega = 0.65$.

Some discrepancies between the tight-binding and photonic models can be associated with a finite thickness of the dielectric slab and electromagnetic interactions beyond nearest neighbors. For instance, in the tight-binding model including only nearest-neighbor interaction, the spectrum of bulk dimerized graphene is symmetric about zero energy. The simulated photonic band structure does not exhibit this property. Similarly, incorporating next-nearest-neighbor interactions in the tight-binding model lifts the symmetry of the spectrum. However, as we have shown, the existence of the triade cavity mode can be inferred within the nearest-neighbor tight-binding approximation.

Remarkably, circularly polarized sources operating at frequencies within the minigap can be employed to selectively excite corners of the cavity, see Fig. S5. This directional coupling stems from the chirality of the evanescent edge states [see Figs. S5(a,b)]. In turn, individual corner modes emit beams with singularities in the far field.

For a large triangular cavity where the corners are weakly coupled, the dependence of the triade mode frequency on TBM parameters t/M is shown in Fig. S6. It has two asymptotics: given by expression Eq. (S22) for small t, and

$$\omega(t \to 2M) = M - \Delta t + C\frac{\Delta t}{2}\left[1 - \frac{\Delta t}{M} + \frac{\Delta t^2}{4M^2}\right] \tag{S28}$$

for t approaching $2M$, where $\Delta t = 2M - t$, and coefficient $C \sim 0.5$. The range covered by the edge state dispersion $\omega = t - \sqrt{M^2 + 4t^2\cos^2(k_x a/2)}$ at fixed t/M is defined by the minimum $\omega_{\min} = t - \sqrt{M^2 + 4t^2}$ and maximum $\omega_{\max} = t - |M|$ values also plotted in Fig. S6.

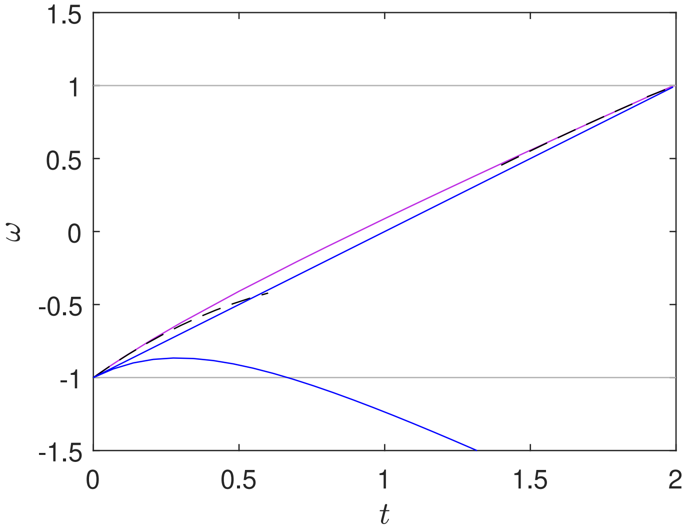

FIG. S6: Dependence of the corner mode frequency on TBM parameters (violet solid line). The cavity side length was taken 32 dimers, the effective mass was fixed as $M = 1$. Black dashed curves are plotted using analytical approximations (S22) and (S28). Horizontal grey lines mark the bulk band edges $\omega = \pm M$. Blue curves trace minimum and maximum values of frequencies of the edge states at a straight zigzag interface.

II. Continuum model of valley-Hall insulators

By substituting $\delta k_{x,y} = -i\partial_{x,y}$, into Eq. (S7), the evolution equations for the two-component wavefunction are transformed to $(2+1)$-dimensional Dirac model

$$\begin{aligned} i\frac{\partial \Psi_1}{\partial t} &= -v_D\left(i\partial_x + \partial_y\right)\Psi_2 + M\Psi_1 \\ i\frac{\partial \Psi_2}{\partial t} &= -v_D\left(i\partial_x - \partial_y\right)\Psi_1 - M\Psi_2\,. \end{aligned} \tag{S29}$$

For propagating bulk modes $e^{-i\omega t + i\delta k_x x + i\delta k_y y}$ in a homogeneous medium with a constant mass, the reduced two-band model (S7) gives the (hyperbolic) dispersion relations for two bands:

$$\omega_\pm(\delta\boldsymbol{k}) = \pm\sqrt{v_D^2(\delta k_x^2 + \delta k_y^2) + M^2}\,, \tag{S30}$$

where $\delta k^2 = \delta k_x^2 + \delta k_y^2$.

A. Valley-Hall edge states in the continuum Dirac model

In the framework of the continuum Dirac model, we next derive dispersion of valley-Hall edge states at the domain walls created by mass inversion. We first determine the effective boundary conditions based on the symmetry of equations. If we consider a straight interface along x axis, owing to the translational invariance, the momentum k_x is a good quantum number, whereas k_y should be replaced by the real-space derivative $-i\partial_y$. Accepting the ansatz $\Psi_{1,2} = \psi_{1,2}(y)e^{-i\omega t + i\delta k_x x}$, equations (S29) are rewritten as

$$\begin{aligned} (-\omega + M(y))\psi_1(y) + (\delta k_x - \partial_y)\psi_2(y) = 0 \\ (\omega + M(y))\psi_2(y) + (-\delta k_x - \partial_y)\psi_1(y) = 0\,. \end{aligned}$$

In the domain-wall problem with the mass inversion $M(y > 0) = M = \text{const}$ and $M(y < 0) = -M$, that leads to two sets of coupled equations in positive $y > 0$ and negative $y < 0$ half-spaces, which differ by the replacement $M \to -M$:

$$\begin{aligned} (-\omega + M)\psi_1(y > 0) + (\delta k_x - \partial_y)\psi_2(y > 0) = 0 \\ (\omega + M)\psi_2(y > 0) + (-\delta k_x - \partial_y)\psi_1(y > 0) = 0 \end{aligned}$$

$$(-\omega - M)\psi_1(y<0) + (\delta k_x - \partial_y)\psi_2(y<0) = 0$$
$$(\omega - M)\psi_2(y<0) + (-\delta k_x - \partial_y)\psi_1(y<0) = 0$$

Hence, the solution satisfies $\psi_1(y<0) = \psi_2(-y)e^{i\varphi}$, $\psi_2(y<0) = \psi_1(-y)e^{i\varphi}$. The continuity boundary condition $\psi_{1,2}(-0) = \psi_{1,2}(+0)$ yields $\psi_1(0)e^{-i\varphi} = \psi_2(0)$, $\psi_2(0)e^{i\varphi} = \psi_1(0)$, $e^{2i\varphi} = 1$, $\varphi = [0, \pi]$. Thus, we can restrict our consideration to the half-space $y > 0$ imposing the effective boundary condition which relates the two components of the spinor as

$$\psi_1(0) = \pm\psi_2(0), \tag{S31}$$

suggesting that the wavefunction is either symmetric or antisymmetric. This is consistent with global parity symmetry of the system with respect to the interface.

For the half-space $y > 0$, we then use the ansatz $\exp(i\delta k_x x - \varkappa y)$, $\delta k_y = i\varkappa$, $\varkappa > 0$ for the edge-bound solution and formally utilize the effective boundary condition (S31) at $y = 0$, that leads to

$$\frac{\psi_1(0)}{\psi_2(0)} = \frac{v_D(\pm\delta k_x + \varkappa)}{\omega - M} = \pm 1, \tag{S32}$$

where the decay factor $\varkappa = \sqrt{v_D^2\delta k_x^2 + M^2 - \omega^2}/v_D$. The dispersion of the edge state near the Dirac point is found to be linear, with $\varkappa = |M|/v_D$. For positive mass $M > 0$, we get $\omega_{K_\pm} = \mp v_D\delta k_x$ (opposite signs in different valleys imply opposite group velocities) and spinor $[1, \mp 1]$, while for negative mass $M < 0$: $\omega_{K_\pm} = \pm v_D\delta k_x$ and spinor $[1, \pm 1]$.

The correspondence between the continuum and and tight-binding models is recovered through

$$\frac{\psi_1(II)}{\psi_2(II)} = \frac{\psi_1(I)}{\psi_2(I)} = \pm 1 = \frac{a_I}{b_I} = \frac{a_{II}}{b_{II}}. \tag{S33}$$

B. Topological cavities

We consider a topological cavity in the geometry, where a circular domain $\rho < \mathcal{R}$ with negative mass $(-M)$ is surrounded by the insulator with the positive mass $M > 0$.

The recovered coupled equations (S29) can be formulated in the polar coordinate system (ρ, φ) for two valley blocks

$$i\frac{\partial\Psi_1}{\partial t} = \mp v_D e^{\mp i\varphi}\left(i\frac{\partial}{\partial\rho} \pm \frac{1}{\rho}\frac{\partial}{\partial\varphi}\right)\Psi_2 + M\Psi_1\,, \tag{S34a}$$
$$i\frac{\partial\Psi_2}{\partial t} = \mp v_D e^{\pm i\varphi}\left(i\frac{\partial}{\partial\rho} \mp \frac{1}{\rho}\frac{\partial}{\partial\varphi}\right)\Psi_1 - M\Psi_2\,. \tag{S34b}$$

System (S34) possesses solutions with harmonic time dependence and radial symmetry

$$\begin{pmatrix}\Psi_1\\ \Psi_2\end{pmatrix}(\rho,\varphi,t;m) = \begin{pmatrix}\psi_1\\ \psi_2\end{pmatrix}e^{-i\omega t} = \begin{pmatrix}u e^{im\varphi}\\ iv e^{i(m+1)\varphi}\end{pmatrix}e^{-i\omega t} \tag{S35}$$

where $m = 0, \pm 1, \pm 2...$ is the azimuthal number. The eigenproblem in the polar coordinate system reads

$$\omega\begin{pmatrix}\psi_1\\ \psi_2\end{pmatrix} = \begin{pmatrix} M & \mp i e^{\mp i\varphi}\left(\frac{\partial}{\partial\rho} \mp \frac{i}{\rho}\frac{\partial}{\partial\varphi}\right)\\ \mp i e^{\pm i\varphi}\left(\frac{\partial}{\partial\rho} \pm \frac{i}{\rho}\frac{\partial}{\partial\varphi}\right) & -M\end{pmatrix}\begin{pmatrix}\psi_1\\ \psi_2\end{pmatrix} \tag{S36}$$

The cavity's spectrum yielded by Eqs. (S36) is discrete and contains edge states that occur in pairs with opposite pseudospins.

Radial functions u, v for the K_+ valley obey the coupled ordinary differential equations

$$\left(\frac{d}{d\rho} + \frac{m+1}{\rho}\right)v + (M - \omega)u = 0\,, \tag{S37a}$$
$$\left(\frac{d}{d\rho} - \frac{m}{\rho}\right)u + (M + \omega)v = 0\,. \tag{S37b}$$

Plugging u from Eq. (S37b) into Eq. (S37a), we obtain a second-order differential equation for v:

$$\frac{d^2v}{d\rho^2} + \frac{1}{\rho}\frac{dv}{d\rho} + \left((\omega^2 - M^2) - \frac{(m+1)^2}{\rho^2}\right) v = 0\,. \tag{S38}$$

Eq. (S38) is simply the Bessel differential equation, whose solution for $\omega^2 < M^2$ are modified Bessel functions (the Bessel functions for complex arguments): $v = CI_{m+1}(\sqrt{M^2-\omega^2}\rho)$ for the region containing $\rho = 0$, and $v = AK_{m+1}(\sqrt{M^2-\omega^2}\rho)$ outside the circle. The ratio of coefficients C/A and frequency ω are found from the continuity boundary condition at $\rho = \mathcal{R}$:

$$C\left(\frac{I_{m+1}(\sqrt{M^2-\omega^2}\mathcal{R})}{\frac{1}{\omega - M}\left(\frac{d}{d\rho}+\frac{m+1}{\rho}\right) I_{m+1}(\sqrt{M^2-\omega^2}\rho)\Big|_{\rho=\mathcal{R}}}\right) = A\left(\frac{K_{m+1}(\sqrt{M^2-\omega^2}\mathcal{R})}{\frac{1}{\omega + M}\left(\frac{d}{d\rho}+\frac{m+1}{\rho}\right) K_{m+1}(\sqrt{M^2-\omega^2}\rho)\Big|_{\rho=\mathcal{R}}}\right) \tag{S39}$$

Thus, the dispersion relation is given by the implicit relation

$$(\omega - M)I_{m+1}(\sqrt{M^2-\omega^2}\mathcal{R})\left(\frac{d}{d\rho}+\frac{m+1}{\rho}\right)K_{m+1}(\sqrt{M^2-\omega^2}\rho)\Big|_{\rho=\mathcal{R}} = (\omega + M)K_{m+1}(\sqrt{M^2-\omega^2}\mathcal{R})\left(\frac{d}{d\rho}+\frac{m+1}{\rho}\right)I_{m+1}(\sqrt{M^2-\omega^2}\rho)\Big|_{\rho=\mathcal{R}}\,. \tag{S40}$$

For the cavities of radius $\mathcal{R}$ much larger than the decay length of the states into the bulk insulator, we can exploit the following asymptotics in Eq. (S40): $I_{m+1} \approx \frac{e^{\varkappa\rho}}{\sqrt{2\pi\varkappa\rho}}$, $K_{m+1} \approx \sqrt{\frac{\pi}{2}}\frac{e^{-\varkappa\rho}}{\sqrt{\varkappa\rho}}$. With this approximation, we obtain the discrete spectrum $\omega(m) = -\frac{v_D}{\mathcal{R}}\left(\frac{1}{2} \pm m\right)$, $\varkappa = |M|/v_D$. In the limit $\varkappa\mathcal{R} \gg 1$, the problem is reduced to the quasi-planar interface considered above by replacing $\frac{m}{\mathcal{R}}$ with a wavenumber δk_x. This suggests that for the modes strongly localized in the vicinity of the boundary $\rho = \mathcal{R}$ we can use analytical solutions obtained above for the planar geometry. Note, in the open system the modes propagating along the domain wall are leaky. With the cavity's size and radiative losses increasing, the spectrum tends to the continuum limit.

C. Bound-state formation at the domain-wall corner

We perform the Taylor-series expansion of the edge state dispersion from Eq. (S14)

$$\omega = t - \sqrt{M^2 + 4t^2\cos^2(k_x a/2)} \tag{S41}$$

in the vicinity of the wavenumber $\bar{K}$:

$$k_x = \bar{K} + \tilde{k}\,,$$
$$\omega = \bar{\omega} + \frac{d\omega}{dk_x}\tilde{k} + \frac{1}{2}\frac{d^2\omega}{dk_x^2}\tilde{k}^2\,.$$

Accepting the monochromatic $\exp(i\omega t)$ process and substituting operators

$$\Delta\omega = \omega - \bar{\omega} = i\frac{\partial}{\partial t}\,,$$
$$\tilde{k} = -i\frac{\partial}{\partial x}\,,$$
$$\tilde{k}^2 = -\frac{\partial^2}{\partial x^2}$$

into the dispersion relation (S41), we then recover the equation for the slowly varying field amplitude

$$i\frac{\partial \mathcal{A}}{\partial t} + iv_g\frac{\partial \mathcal{A}}{\partial x} - \alpha\frac{\partial^2 \mathcal{A}}{\partial x^2} = 0\,, \tag{S42}$$

where $v_g = \left.\frac{d\omega}{dk_x}\right|_{k_x=K}$ is the group velocity, $\alpha = -\frac{1}{2}\left.\frac{d^2\omega}{dk_x^2}\right|_{k_x=K}$ is the second-order dispersion coefficient. For the localised solution at rest $v_g = 0$, hence, $\bar{K}a = \pi$. In this case $\alpha = \frac{a^2t^2}{2|M|}$, $\bar{\omega} = t - |M|$, and Eq. (S42) transforms into a parabolic equation,

$$i\frac{\partial \mathcal{A}}{\partial t} - \alpha\frac{\partial^2 \mathcal{A}}{\partial x^2} = 0\,. \tag{S43}$$

In this setting, the corner of the domain wall plays the role of a trapping potential in Eq. (S43). Next, we denote by s the coordinate along the domain wall path [see Fig. S1(b)] and rewrite Eq. (S43) introducing the potential $U(s)$ and assuming $\mathcal{A} = \bar{\mathcal{A}}(s)e^{-i\Delta\omega t}$

$$\frac{\partial^2 \bar{\mathcal{A}}}{\partial s^2} - \frac{\Delta\omega}{\alpha}\bar{\mathcal{A}} + U(s)\bar{\mathcal{A}} = 0\,, \tag{S44}$$

where $U(s)$ is given by

$$U(s) = \frac{3}{2\pi\mathcal{R}}\left\{\begin{array}{l} 1, \ \text{for } |s| \le \frac{\pi\mathcal{R}}{3} \\ 0, \ \text{for } |s| > \frac{\pi\mathcal{R}}{3} \end{array}\right\} \times \frac{2}{a}A_U\,, \tag{S45}$$

with the potential amplitude $A_U \equiv A_U\left(\frac{\Delta t}{2M}\right)$ being a function of the parameter $\frac{\Delta t}{2M}$; $\mathcal{R}$ is the radius of the path curvature. Here, we specifically consider the regime $t \to 2M$, when the edge state dispersion curve nearly merges to the upper bulk band at $k_xa = \pi$. At vanishing $\mathcal{R} \to 0$ the potential turns to the Dirac delta function $U(s) = \delta(s)\frac{2}{a}A_U$ and from Eq. (S44) we find the decay rate $\varkappa = \sqrt{\frac{\Delta\omega}{\alpha}} = \frac{1}{a}A_U$, and the frequency of the trapped state

$$\omega \equiv \bar{\omega} + \Delta\omega = t - M + \Delta\omega = M - \Delta t + 2MA_U^2\left[1 - \frac{\Delta t}{M} + \frac{\Delta t^2}{4M^2}\right]\,. \tag{S46}$$

This formula can be used to fit the numerically calculated dispersion of corner modes choosing $A_U = \sqrt{\frac{C}{2}}\sqrt{\frac{\Delta t}{2M}}$, that coincides with Eq. (S28) .

III. Effective electromagnetic Hamiltonian from the plane wave expansion method

We derive the effective Hamiltonian in the vicinity of Dirac points for a 2D honeycomb photonic crystal slab with air holes by using the plane wave expansion method. Accepting the time dependency $\sim \exp(-i\omega t)$, we start with Maxwell's equations,

$$\begin{aligned} \nabla \times \mathbf{E} &= ik_0\mu\mathbf{H}\,, \\ \nabla \times \mathbf{H} &= -ik_0\varepsilon\mathbf{E}\,, \end{aligned} \tag{S47}$$

where $k_0 = \omega/c$ is the wavenumber in free space, ω is the angular frequency, and c is the speed of light. Material response is assumed to be described by effective parameters of a two-dimensional medium, namely, a scalar dielectric permittivity $\varepsilon(x, y)$ and the constant magnetic permeability $\mu = 1$.

Implying $\partial_z = 0$, from Eq. (S47) we obtain the governing equation for the $H_z(x, y)$ electric field component of TE polarization

$$k_0^2H_z + \frac{1}{\varepsilon}\left(\frac{\partial^2}{\partial x^2} + \frac{\partial^2}{\partial y^2}\right)H_z = 0\,. \tag{S48}$$

Here, we assume in-plane propagation. Given the crystal periodicity, we apply Bloch theorem and expand the field H_z and the permittivity in Fourier series as follows,

$$H_z = \sum_G H_{\mathbf{G}}\, e^{i(\mathbf{G}+\mathbf{q})\cdot\mathbf{r}}\,, \tag{S49}$$

$$\varepsilon^{-1} = \sum_G \tilde{\varepsilon}_{\mathbf{G}}\, e^{i\mathbf{G}\cdot\mathbf{r}}\,, \tag{S50}$$

where $\mathbf{G}$ and $\mathbf{G}'$ denote reciprocal lattice vectors, $\mathbf{q}$ is quasi-momentum. The length of all reciprocal lattice vectors $\mathbf{G}_i$ is equal to $G = 4\pi/(\sqrt{3}\,a)$, where a is a lattice constant of the crystal. Substituting Eqs. (S49), (S50) into Eq. (S48), we get a set of linear equations for the Fourier components of the field

$$k_0^2 H_{\mathbf{G}} - \sum_{G'} \tilde{\varepsilon}_{\mathbf{G}-\mathbf{G}'} |\mathbf{q}+\mathbf{G}'|^2 H_{\mathbf{G}'} = 0\,. \tag{S51}$$

We are now interested in dispersion of the modes near the Dirac points being the corners of the crystal Brillouin zone, which correspond to the Bloch wavevectors $\mathbf{K}_\pm = K(\pm1,0,0)$, where $K = \dfrac{4\pi}{3a}$. Hence, for the K_+ (K_-) valleys $\mathbf{q}+\mathbf{G} = \mathbf{K}_\pm + \delta\mathbf{k} + \mathbf{G} \equiv \mathbf{k} + \delta\mathbf{k}$, where $\delta\mathbf{k}$ is a small detuning. We truncate the basis to the first three plane waves with the wavevectors $\mathbf{k}_{1,2,3} = \mathbf{K}_\pm + \mathbf{G}_{0,1,2}$ each rotated by $2\pi/3$ with respect to one another and corresponding to the reciprocal lattice vectors $\mathbf{G}_0 = (0,0)$, $\mathbf{G}_1 = K\left(\mp\frac{3}{2}, -\frac{\sqrt{3}}{2}\right)$, $\mathbf{G}_2 = K\left(\mp\frac{3}{2}, \frac{\sqrt{3}}{2}\right)$. Thus, to describe the formation of the bands, we leave only the leading contributions from three Γ points nearest to $K_\pm$ with $\mathbf{k}_1 = (\pm K, 0)$, $\mathbf{k}_2 = K\left(\mp\frac{1}{2}, -\frac{\sqrt{3}}{2}\right)$, $\mathbf{k}_3 = K\left(\mp\frac{1}{2}, \frac{\sqrt{3}}{2}\right)$. Next, we apply $k\cdot p$ approximation, keeping only the first order in $\delta\mathbf{k}$, $(\mathbf{k}_j + \delta\mathbf{k})^2 \approx \mathbf{k}_j^2 + 2\mathbf{k}_j\cdot\delta\mathbf{k} = K^2 + 2\mathbf{k}_j\cdot\delta\mathbf{k}$. Neglecting the terms of higher orders of smallness, we obtain a 3×3 set of equations

$$k_0^2 \begin{pmatrix} H_1 \\ H_2 \\ H_3 \end{pmatrix} = \left[K^2 \begin{pmatrix} \tilde{\varepsilon}_0 & \tilde{\varepsilon}_{12} & \tilde{\varepsilon}_{13} \\ \tilde{\varepsilon}_{12}^* & \tilde{\varepsilon}_0 & \tilde{\varepsilon}_{23} \\ \tilde{\varepsilon}_{13}^* & \tilde{\varepsilon}_{23}^* & \tilde{\varepsilon}_0 \end{pmatrix} + 2\delta\mathbf{k}\cdot \begin{pmatrix} \mathbf{k}_1\tilde{\varepsilon}_0 & \mathbf{k}_2\tilde{\varepsilon}_{12} & \mathbf{k}_3\tilde{\varepsilon}_{13} \\ \mathbf{k}_1\tilde{\varepsilon}_{12}^* & \mathbf{k}_2\tilde{\varepsilon}_0 & \mathbf{k}_3\tilde{\varepsilon}_{23} \\ \mathbf{k}_1\tilde{\varepsilon}_{13}^* & \mathbf{k}_2\tilde{\varepsilon}_{23}^* & \mathbf{k}_3\tilde{\varepsilon}_0 \end{pmatrix}\right] \begin{pmatrix} H_1 \\ H_2 \\ H_3 \end{pmatrix}. \tag{S52}$$

The Fourier coefficients comprising the above system are defined as

$$\tilde{\varepsilon}_{ij} = \frac{1}{S_0} \int\limits_{\text{u.c.}} \varepsilon^{-1}(x,y)\, e^{-i(\mathbf{G}_i-\mathbf{G}_j)\cdot\mathbf{r}}\, d^2\mathbf{r}_\perp\,, \tag{S53}$$

where $S_0 = a^2\sqrt{3}/2$ is the area of the structure unit cell, and the index $(\mathbf{G}_i - \mathbf{G}_j) \equiv \mathbf{G}_{ij}$ is abbreviated as ij. In particular, coefficients $\tilde{\varepsilon}_{00,11,22} \equiv \tilde{\varepsilon}_0$ imply the averaged over the unit cell spatial distribution. Additionally, we make use of a honeycomb symmetry of the lattice.

We consider the case of parity breaking via *dimerization*, when two rods A and B in the bipartite unit cell of the honeycomb lattice have equal dielectric permittivities $\varepsilon_{\mathrm{A}} = \varepsilon_{\mathrm{B}} = \varepsilon_1$ but slightly different radii $r_{\mathrm{A}} = r_0 - \Delta r$, $r_{\mathrm{B}} = r_0 + \Delta r$. Accordingly, we write the spatial distribution of the permittivity as

$$\varepsilon^{-1}(\mathbf{r}_\perp) = \varepsilon_0^{-1} + (\varepsilon_1^{-1} - \varepsilon_0^{-1})\Pi_{r_0-\Delta r}(\mathbf{r}_\perp - \mathbf{v}_1) + (\varepsilon_1^{-1} - \varepsilon_0^{-1})\Pi_{r_0+\Delta r}(\mathbf{r}_\perp + \mathbf{v}_1), \tag{S54}$$

where function $\Pi_{r_0}(\mathbf{r}_\perp)$ is a rectangular functions of the width $2r_0$, the nearest-neighbor vector $\mathbf{v}_1 = (0,1)\,\dfrac{a_0}{2}$, $a_0 = \dfrac{a}{\sqrt{3}}$ is a distance between neighboring cylinders A and B. Values ε_0^{-1} and ε_1^{-1} correspond to the environment and interiors of cylinders. Thereby, coefficients (S53) take the form

$$\tilde{\varepsilon}_0 \approx \varepsilon_0^{-1} + 2f_0(\varepsilon_1^{-1} - \varepsilon_0^{-1})\,,\ i=j \tag{S55a}$$

$$\tilde{\varepsilon}_{ij} \approx \tilde{\varepsilon}_1 2\cos\varphi_{ij} + \Delta\tilde{\varepsilon} 2i\sin\varphi_{ij}\,,\ i\neq j\,, \tag{S55b}$$

where filling factor $f_0 = \pi r_0^2/S_0$, angle $\varphi_{ij} = (\mathbf{G}_{ij}\cdot\mathbf{v}_1)$, and we denote $\tilde{\varepsilon}_1 = (\varepsilon_1^{-1} - \varepsilon_0^{-1})\dfrac{2f_0}{Gr_0}\,(J_1(Gr_0) + J_1' G\Delta r)$, $\Delta\tilde{\varepsilon} = (\varepsilon_1^{-1} - \varepsilon_0^{-1})\dfrac{2f_0\Delta r}{Gr_0^2}\,J_1(Gr_0)$, where J_1 and J_1' is the Bessel function of the first kind and its derivative, respectively. Calculating the angles, we get $\varphi_{12} = \varphi_{31} = -\varphi_{21} = -\varphi_{13} = \pi/3$, $\varphi_{23} = -2\pi/3 = -\varphi_{32}$.

Therefore, from Eq. (S52) we obtain 3×3 eigenvalue problem

$$k_0^2 \begin{pmatrix} H_1 \\ H_2 \\ H_3 \end{pmatrix} = \Bigg[K^2 \begin{pmatrix} \tilde{\varepsilon}_0 & \tilde{\varepsilon}_1 & \tilde{\varepsilon}_1 \\ \tilde{\varepsilon}_1 & \tilde{\varepsilon}_0 & -\tilde{\varepsilon}_1 \\ \tilde{\varepsilon}_1 & -\tilde{\varepsilon}_1 & \tilde{\varepsilon}_0 \end{pmatrix} + iK^2 \Delta\tilde{\varepsilon}\sqrt{3} \begin{pmatrix} 0 & 1 & -1 \\ -1 & 0 & -1 \\ 1 & 1 & 0 \end{pmatrix} \pm K\delta k_x \begin{pmatrix} 2\tilde{\varepsilon}_0 & -\tilde{\varepsilon}_1 & -\tilde{\varepsilon}_1 \\ 2\tilde{\varepsilon}_1 & -\tilde{\varepsilon}_0 & \tilde{\varepsilon}_1 \\ 2\tilde{\varepsilon}_1 & \tilde{\varepsilon}_1 & -\tilde{\varepsilon}_0 \end{pmatrix} + K\delta k_y \sqrt{3} \begin{pmatrix} 0 & -\tilde{\varepsilon}_1 & \tilde{\varepsilon}_1 \\ 0 & -\tilde{\varepsilon}_0 & -\tilde{\varepsilon}_1 \\ 0 & \tilde{\varepsilon}_1 & \tilde{\varepsilon}_0 \end{pmatrix} \Bigg] \begin{pmatrix} H_1 \\ H_2 \\ H_3 \end{pmatrix}, \quad (S56)$$

or equivalently, $k_0^2 \boldsymbol{H} = \hat{H}_1 \boldsymbol{H}$, where the column-vector $\boldsymbol{H} = (H_{\mathbf{G}_0}, H_{\mathbf{G}_1}, H_{\mathbf{G}_2})^{\mathrm{T}}$, and the 3×3 matrix $\hat{H}_1$ of this system is Hermitian. After performing a unitary transformation $\hat{H} = U \hat{H}_1 U^{-1}$ with the matrix

$$U = \frac{1}{\sqrt{3}} \begin{pmatrix} e^{2i\pi/3} & e^{-i\pi/3} & -1 \\ 1 & e^{-i\pi/3} & e^{i\pi/3} \\ e^{i\pi/3} & e^{-2i\pi/3} & e^{-2i\pi/3} \end{pmatrix}, \quad (S57)$$

we notice that the third row of the transformed matrix describes a nondegenerate singlet TE state, and therefore it can be eliminated. Thus, we recover the following effective massive Dirac Hamiltonian in the subspace of the doublet states for two valleys after anticlockwise rotating the vector $(\delta k_x, \delta k_y)$ by $\frac{4\pi}{3}$,

$$\hat{H}_{K_+(K_-)} = \omega_0 \pm v_D \delta k_x \hat{\sigma}_x + v_D \delta k_y \hat{\sigma}_y + M \hat{\sigma}_z = \begin{pmatrix} \omega_0 + M & v_D(\pm\delta k_x - i\delta k_y) \\ v_D(\pm\delta k_x + i\delta k_y) & \omega_0 - M \end{pmatrix}, \quad (S58)$$

where the unperturbed frequency $\omega_0 = K^2(\tilde{\varepsilon}_0 + \tilde{\varepsilon}_1)$, velocity $v_D = K(\tilde{\varepsilon}_0 + \tilde{\varepsilon}_1)$, and the mass term due to parity breaking (dimerization) $M = 3K^2 \Delta\tilde{\varepsilon}$.

IV. Photonic implementation of staggered graphene: Fabrication recipe

Supplementary Figure S7 illustrates the main stages of the technique used for fabrication of our active metasurface.

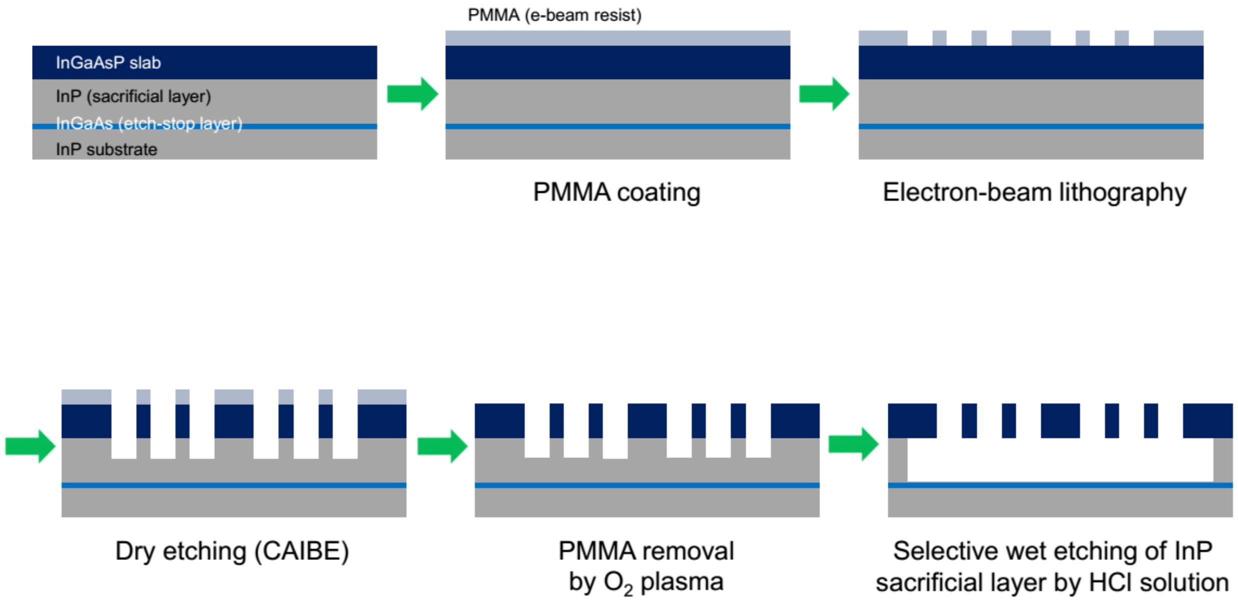

FIG. S7: Schematic of the fabrication procedure.

As mentioned in the main text, the wafer consists of a 250-nm-thick InGaAsP/800-nm-thick InP/100-nm-thick InGaAs/InP substrate that includes three quantum wells with a central emission wavelength of 1.5 mm. We employ

the electron-beam lithography to place a PMMA mask on the InGaAsP slab, and perform a chemically-assisted ion-beam etching (CAIBE) to make holes into the slab. After the PMMA layer is removed by oxygen plasma, the free-standing slab structure is formed by wet-etching the sacrificial InP layer underneath the InGaAsP slab, using a diluted HCl:H2O (3:1) solution at room temperature.
